# Exploring the relationship between gut microbiota and breast cancer risk in European and East Asian populations using Mendelian randomization

**DOI:** 10.1186/s12885-024-12721-9

**Published:** 2024-08-08

**Authors:** Wei Lin, Chenghao Gu, Zheyin Chen, Shihang Xue, Haiyan Wu, Liuhai Zeng

**Affiliations:** Xiangshan First People’s Hospital Medical and Health Group, Ningbo, Zhejiang Province 315700 China

**Keywords:** Gut microbiota, Breast cancer, Mendelian randomization, Europeans, East Asians

## Abstract

**Background:**

Several studies have explored the potential link between gut microbiota and breast cancer; nevertheless, the causal relationship between gut microbiota and breast cancer remains unclear.

**Methods:**

We utilized summary statistics from genome-wide association studies (GWAS) of the gut microbiome from the MiBioGen project with summary data from GWAS on breast cancer from the FinnGen consortium and the IEU database, with the IEU data sourced from the Biobank Japan. Preliminary statistical analyses were conducted using inverse variance weighting (IVW), supplemented by various sensitivity analysis methods, including MR-Egger regression, weighted median, weighted mode, simple median, and simple mode, to ensure the robustness of our findings. Heterogeneity and pleiotropy were assessed to avoid misleading conclusions caused by unconsidered confounders or non-specific effects of genetic variants, ensuring that the results reflect a genuine causal relationship.

**Results:**

In European populations, four types of gut microbiota were associated with breast cancer. The genus *Erysipelatoclostridium* was positively associated with the risk of breast cancer, with an odds ratio (OR) of 1.21 (95% confidence interval [CI] 1.083–1.358), false discovery rate (FDR) = 0.0039. The class Coriobacteriia, order Coriobacteriales, and family Coriobacteriaceae, which belong to the same phylogenetic system, showed a consistent inversely association with breast cancer risk, with an OR of 0.757 (95% CI 0.616–0.930), FDR = 0.0281. In East Asian populations, three types of gut microbiota were related to breast cancer. The *Eubacterium ruminantium* group was positively associated with breast cancer risk, with an OR of 1.259 (95% CI 1.056–1.499), FDR = 0.0497. The families Porphyromonadaceae and Ruminococcaceae were inversely associated with breast cancer risk, with ORs of 0.304 (95% CI 0.155–0.596), FDR = 0.0005, and 0.674 (95% CI 0.508–0.895), FDR = 0.03173, respectively. However, these two taxa had limited instrumental variables, restricting the statistical power and potentially affecting the interpretation of the results.

**Conclusion:**

This MR analysis demonstrated a probable causal link between specific gut microbiota and breast cancer. This study, through Mendelian randomization analysis comparing European and East Asian populations, reveals that gut microbiota may influence breast cancer risk differently across populations, providing potential directions for developing targeted prevention and treatment methods.

## Introduction

According to the International Agency for Research on Cancer of the World Health Organization in 2021, the incidence of breast cancer has surpassed that of lung cancer, becoming the most common cancer worldwide [1]. The pathogenesis of breast cancer is complex, with numerous identified risk factors, including genetic mutations (BRCA1, BRCA2) [2], lifestyle factors (alcohol consumption, smoking, high-fat diet, lack of exercise, intake of exogenous hormones such as oral contraceptives) [3], reproductive history (early menarche, late menopause, nulliparity or late childbearing) [4], obesity [5], and radiation exposure [6]. Given the multifactorial nature of this disease, there are likely unknown factors involved in breast cancer development.

The gut microbiota is the complex community of microorganisms residing in the human gastrointestinal tract, which directly or indirectly participate in tumorigenesis and progression through mechanisms such as influencing host inflammatory responses, promoting the formation of the tumor microenvironment, and manipulating tumor cell signaling pathways [7–9]. For example, dysbiosis is characterized by an increase in harmful bacteria and a decrease in beneficial bacteria and may raise the risk of breast cancer [10]. Some carcinogenic substances produced by some gut microbes, including bile acid metabolic products, might affect distant breast tissues via circulation, promoting cell proliferation and apoptosis [11]. Certain bacteria within the gut microbiome, known as the “estrobolome,” possess genes for metabolizing estrogens. The activity of these bacteria may influence the risk of estrogen receptor-positive breast cancer in postmenopausal women [12]. Studies proposed a mechanism known as the “gut-breast axis,” where gut microbiota could affect breast cancer development by transferring immune cells to lymph nodes, which then migrate to the breast via the bloodstream or lymphatic system [8].

The gut microbiome is a complex ecosystem comprising trillions of microorganisms. Considering its role in human health and disease, in-depth studies hold promise for opening avenues for treating and preventing various diseases [13]. In recent years, there has been growing interest in the relationship between the gut microbiome and breast cancer. Goedert et al. identified distinctive compositional differences between untreated breast cancer patients and healthy individuals, with characteristic microbiota including Clostridiaceae, Faecalibacterium, and Ruminococcaceae [14]. Terrisse et al. compared healthy individual samples and found that *Bacteroides uniformis*, *Clostridium bolteae*, and *Bilophila wadsworthia* were associated with poorer breast cancer outcomes [15]. However, some studies suggest that there is not a significant difference in the gut microbiota between breast cancer and non-breast cancer patients [16].

Research provides a preliminary understanding of the relationship between gut microbiota and breast cancer; however, it remains primarily confined to observational studies [17–19]. Although observational studies can reveal potential associations, their conclusions are often affected by potential confounders and cannot establish causality. Mendelian randomization (MR) utilizes genetic variants as instrumental variables and reduces biases inherent in traditional observational studies, making it possible to infer causality where randomized controlled trials are not feasible. Therefore, we employed MR techniques using genome-wide association studies (GWAS) summary statistics to explore the potential causal relationship between the gut microbiome and the risk of developing breast cancer.

## Methods

### Study design

**Fig. 1:**
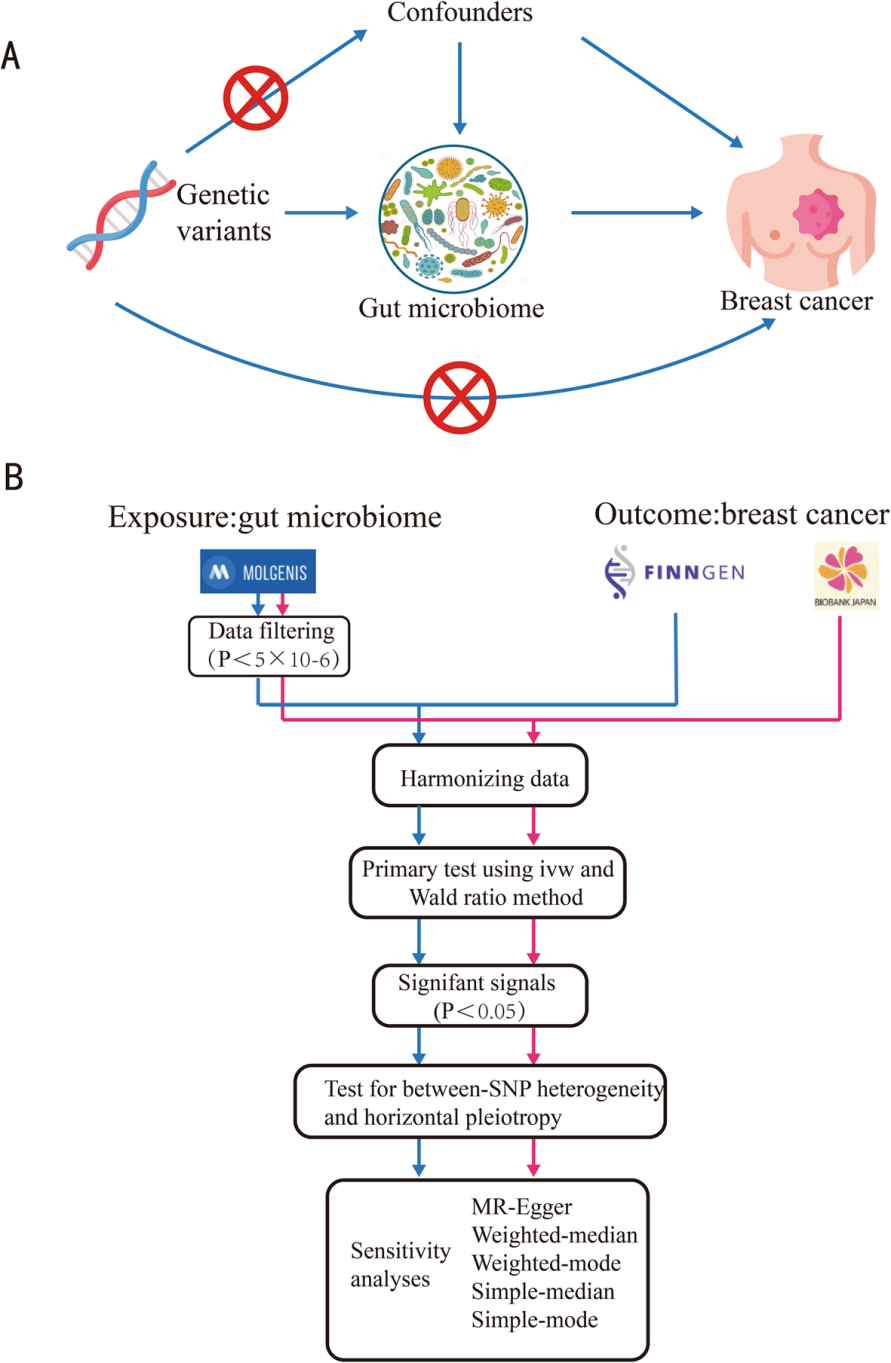
Study Design and Flowchart. **A** The basic schema of Mendelian Randomization (MR) analysis, where we designated gut microbiota as the exposure and breast cancer as the outcome. Arrow symbols are used to denote the assumptions of Mendelian Randomization. **B** Data analysis: We conducted two independent MR analyses using the same exposure data but different outcome data (i.e., breast cancer data from FinnGen and Biobank Japan)

STROBE-MR guidelines were used in the design of this study, specifically developed for two-sample Mendelian randomization [20]. The research framework is depicted in Fig. 1A. The gut microbiota served as the exposure, and breast cancer was the outcome. Genetic variants significantly associated with the gut microbiome are selected as instrumental variables (IVs) for investigating the potential causal relationship between gut microbiota and breast cancer using the MR method. MR employs summary data from GWAS to isolate confounding factors, offering a more precise evaluation of causal relationships. Within the MR study framework, IVs must meet three core criteria: (1) be significantly associated with the exposure (relevance criterion); (2) not be associated with any known or unknown confounders (independence criterion); and (3) influence the outcome solely through the exposure and not via any other direct causal pathways (exclusion restriction criterion).

### Source of gut bacteria data

Summary data on gut bacteria were obtained from the genome-wide association meta-analysis conducted by the MiBioGen consortium (https://mibiogen.gcc.rug.nl/), which represents the most extensive study to date on the transgenic genetics of the human gut microbiome. This study compiled data from 18,340 samples across 24 populations from Europe, Africa, Asia, the Middle East, and Latin America, analyzing microbial compositions across multiple variable regions of the 16S rRNA gene, including V1-V2, V3-V4, and V4. DNA was extracted from fecal samples using seven methods, yielding genetic information on 211 types of gut microbes. The data from all samples were normalized to 10,000 reads per sample, and direct classification binning was used to categorize the microbes into five different taxonomic levels: phylum, class, order, family, and genus, based on genetic characteristics [21]. To ensure the study’s comprehensiveness, all identified gut bacterial taxa categories were included, comprising 15 unnamed bacterial taxa and one duplicate bacterial taxon, a total of 211 gut bacterial taxa categories selected as exposure factors for MR analysis.

### Breast cancer data sources

The GWAS data for the European population on breast cancer were sourced from the FinnGen consortium’s R10 release, a large-scale biomedicine project based on the Finnish population, involving 412,181 participants (181,871 males and 230,310 females), with 18,786 breast cancer cases and 182,927 controls. Each GWAS received approval from an ethics review committee, and detailed information on the data can be downloaded from the FinnGen consortium’s official website: https://storage.googleapis.com/finngen-public-data-r10/summary_stats/R10_manifest.tsv/.

For the East Asian population, breast cancer GWAS data were obtained from the Biobank Japan project, the largest known biobank focusing on the East Asian population. From 2003 to 2018, in collaboration with 12 medical institutions, DNA, serum, and clinical information from over 200,000 patients with 47 diseases were collected [22]. Based on these data, researchers identified various genetic variants associated with disease susceptibility and drug responsiveness. The database includes 5,552 breast cancer patients and 89,732 controls, involving 8,872,152 single nucleotide polymorphisms (SNPs). All related GWAS received approval from the ethics committees of the RIKEN Yokohama Institute and the Institute of Medical Science at the University of Tokyo. The diagnosis of primary breast cancer cases was confirmed based on the International Classification of Diseases (ICD) codes applicable during the study period, which includes from the eighth to the tenth editions of the ICD. Detailed information on the data has been included in the publicly accessible IEU database, available for download via the designated dataset (GWAS ID: bbj-a160; https://gwas.mrcieu.ac.uk/).

### Selection of instrumental variables

SNPs strongly associated with the exposure at the genome-wide significance level (*P* < 5 × 10^-6) were selected solely based on their *p*-value. Only the *p*-value was considered in the selection of strongly associated SNPs. To ensure independence, SNPs with high linkage disequilibrium (R^2 < 0.001, distance = 1 MB) were removed. SNPs with fewer than three occurrences and minor allele frequency (MAF) ≤ 0.01 were excluded. We also eliminated SNPs not found in the outcome GWAS dataset, those with inconsistent alleles between exposure and outcome, and palindromic SNPs that could lead to bias.

The F-statistic was calculated to assess potential IV bias, selecting *F* > 10 to ensure weak instrumental variables do not influence causality. The calculation formula is F = R^2^ × (N—1—K) / (1—R^2^) × K, where N represents the sample size of exposure data, K represents the number of IVs, and R^2^ represents the proportion of variance in the exposure variable explained by the selected IVs, with the calculation formula R^2^ = β^2^ / (β2 + SE^2^).

### Statistical analysis

The statistical workflow is depicted in Fig. 1B. After harmonizing the data for exposure and outcome, we conducted MR analysis using the IVW method. Given that IVW provides the most accurate causal effect estimates when all instrumental variables are valid, it was chosen as our primary analysis method. Cochran’s Q test was utilized to assess the consistency of the estimated effects of the selected IVs on the exposure variable, with *p* < 0.05 indicating significant heterogeneity. The MR-Egger intercept was used to detect pleiotropy, with a more significant intercept suggesting more robust evidence of pleiotropy. A zero intercept indicates no pleiotropy, and the *p*-value was used to test whether it significantly differs from zero, with *p* > 0.05 considered insufficient evidence of pleiotropy. Leave-one-out analysis was conducted to evaluate the impact of the removal of individual SNPs on the overall statistical results and to reveal the contribution of specific SNPs to the final analysis outcome. The MR-PRESSO method was applied to detect and remove outliers inconsistent with the predicted effect size or direction. After excluding these outliers, five other MR methods are used for sensitivity analysis to ensure the accuracy and reliability of the final analysis results. These methods include MR-Egger regression, weighted median, weighted mode, simple median, and simple mode. MR-Egger regression was applied to detect and adjust for directional pleiotropy. The weighted median and weighted mode methods can provide robust causal estimates even if up to 50% of the instrumental variables are invalid. The simple median and simple mode methods were also used to ensure the robustness of the results. The combined use of these methods enhances the reliability of our causal inferences. These methods combine IVs ahead of the regression to provide robust causal effect estimates and use these combined IVs to calculate odds ratios (ORs). The effect size of the impact of gut microbiota on breast cancer is expressed as OR and their 95% confidence intervals (CI). The false discovery rate (FDR) was used to adjust for multiple hypothesis testing. All analyses are performed using the “TwoSampleMR,” “MR-PRESSO,” and “Mendelian Randomization” packages in R software (version 4.3.2).

## Results

The MiBioGen study identified 211 gut microbiota taxa belonging to nine phyla, 15 classes, 20 orders, 32 families, and 119 genera.

In the MR analysis of the European population, we identified four categories of gut microbiota significantly associated with the risk of breast cancer, including the class Coriobacteriia, the order Coriobacteriales, the family Coriobacteriaceae, and the genus *Erysipelatoclostridium*. The first three have a hierarchical taxonomic relationship: Coriobacteriia includes the order Coriobacteriales, which belongs to the family Coriobacteriaceae [23]. IVs for these three taxonomic levels, selected based on a statistical threshold of *p*-value less than 5 × 10^–6^, were completely consistent.

**Fig. 2:**
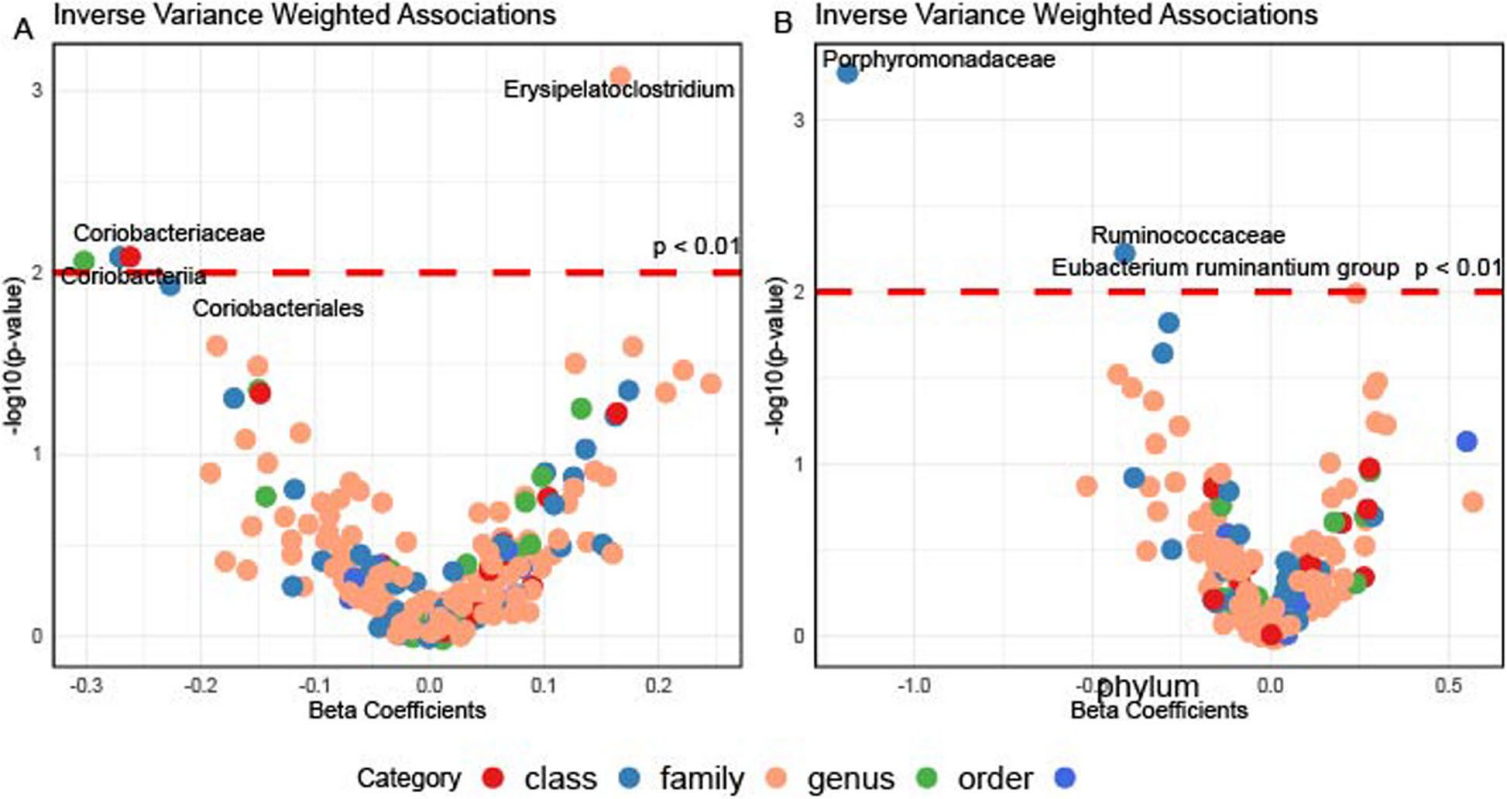
The Association Between Gut Microbiota and Breast Cancer. Subfigure (**A**) and (**B**) show results in Europeans and East Asians, respectively. The red dashed line denotes the statistical significance threshold (i.e., *p* < 0.05). The points were jittered to avoid overlap

In the East Asian population, we found three categories of gut microbiota associated with breast cancer risk (*p* < 0.01), including two families (Porphyromonadaceae and Ruminococcaceae) and one genus (*Eubacterium ruminantium* group) (see Fig. 2B).

These findings were based on the primary MR analysis method (IVW). We conducted further analyses on the gut microbiota, with detailed results in Table 1.

**Table 1:** Mendelian randomization analysis statistics

Populations	Gut microbe	No. of IV	F-statistics	Between-SNP heterogeneity		Horizontal pleiotropy		P (MR-PRESSO global test)
				*Q*-value	*P*-value	Egger intercept	*P*-value	
Europeans	Coriobacteriaceae	5	22.01	2.81	0.589	-0.014	0.757	0.656
	Erysipelatoclostridium	8	24.27	3.05	0.880	0.007	0.726	0.899
	Coriobacteriales	5	22.01	2.81	0.589	-0.014	0.757	0.656
	Coriobacteriia	5	22.01	2.81	0.589	-0.014	0.757	0.656
East Asians	Porphyromonadaceae	2	23.4	0.998	0.318	-	-	-
	Ruminococcaceae	3	23.7	1.64	0.440	-0.112	0.473	-
	Eubacterium ruminantium group	8	22.2	4.55	0.714	0.033	0.352	0.735

Five to eight instrumental variables (IVs) were used for the selected gut microbiota, with an average F-statistic indicating robustness (Table 1). The specific genetic variants are detailed in Table 2. No heterogeneity or horizontal pleiotropy was observed among the SNPs of any gut microbiota (Table 1).

**Table 2:** Specific Genetic Variants Selected

Populations	Gut microbe	ref	alt	SNPs	beta	SE	Pval
Europeans	Erysipelatoclostridium	T	C	rs17804233	0.01	0.011	0.368
		G	A	rs340991	-0.011	0.013	0.385
		G	A	rs4697572	-0.014	0.014	0.321
		T	G	rs58236560	-0.017	0.017	0.334
		G	A	rs622418	-0.013	0.011	0.259
		A	C	rs6474512	-0.031	0.012	0.008
		T	C	rs710230	-0.032	0.022	0.145
		A	G	rs7221249	-0.008	0.011	0.468
	Coriobacteriales	C	T	rs11250875	-0.022	0.014	0.118
		A	G	rs1816223	-0.021	0.014	0.144
		C	T	rs240104	0.019	0.013	0.136
		T	G	rs34739816	-0.042	0.024	0.09
		G	A	rs719099	0.007	0.019	0.722
	Coriobacteriaceae	C	T	rs11250875	-0.022	0.014	0.118
		A	G	rs1816223	-0.021	0.014	0.144
		C	T	rs240104	0.019	0.013	0.136
		T	G	rs34739816	-0.042	0.024	0.09
		G	A	rs719099	0.007	0.019	0.722
	Coriobacteriia	C	T	rs11250875	-0.022	0.014	0.118
		A	G	rs1816223	-0.021	0.014	0.144
		C	T	rs240104	0.019	0.013	0.136
		T	G	rs34739816	-0.042	0.024	0.09
		G	A	rs719099	0.007	0.019	0.722
East Asians	Porphyromonadaceae	C	G	rs10119172	-0.028	0.024	0.245
		A	G	rs17065783	0.087	0.026	0.001
	Ruminococcaceae	T	C	rs17376049	-0.079	0.056	0.158
		C	T	rs2113833	0.081	0.031	0.01
		T	C	rs56199908	0.035	0.049	0.474
	Eubacterium ruminantium group	C	T	rs139749	-0.048	0.022	0.028
		G	A	rs16891896	-0.021	0.028	0.437
		C	T	rs17519472	0.011	0.028	0.703
		A	G	rs2116427	0.009	0.022	0.681
		T	C	rs57340348	0.008	0.037	0.837
		G	A	rs606117	-0.04	0.049	0.415
		G	A	rs7000472	0.036	0.022	0.107
		C	T	rs72836424	-0.051	0.035	0.143

**Fig. 3:**
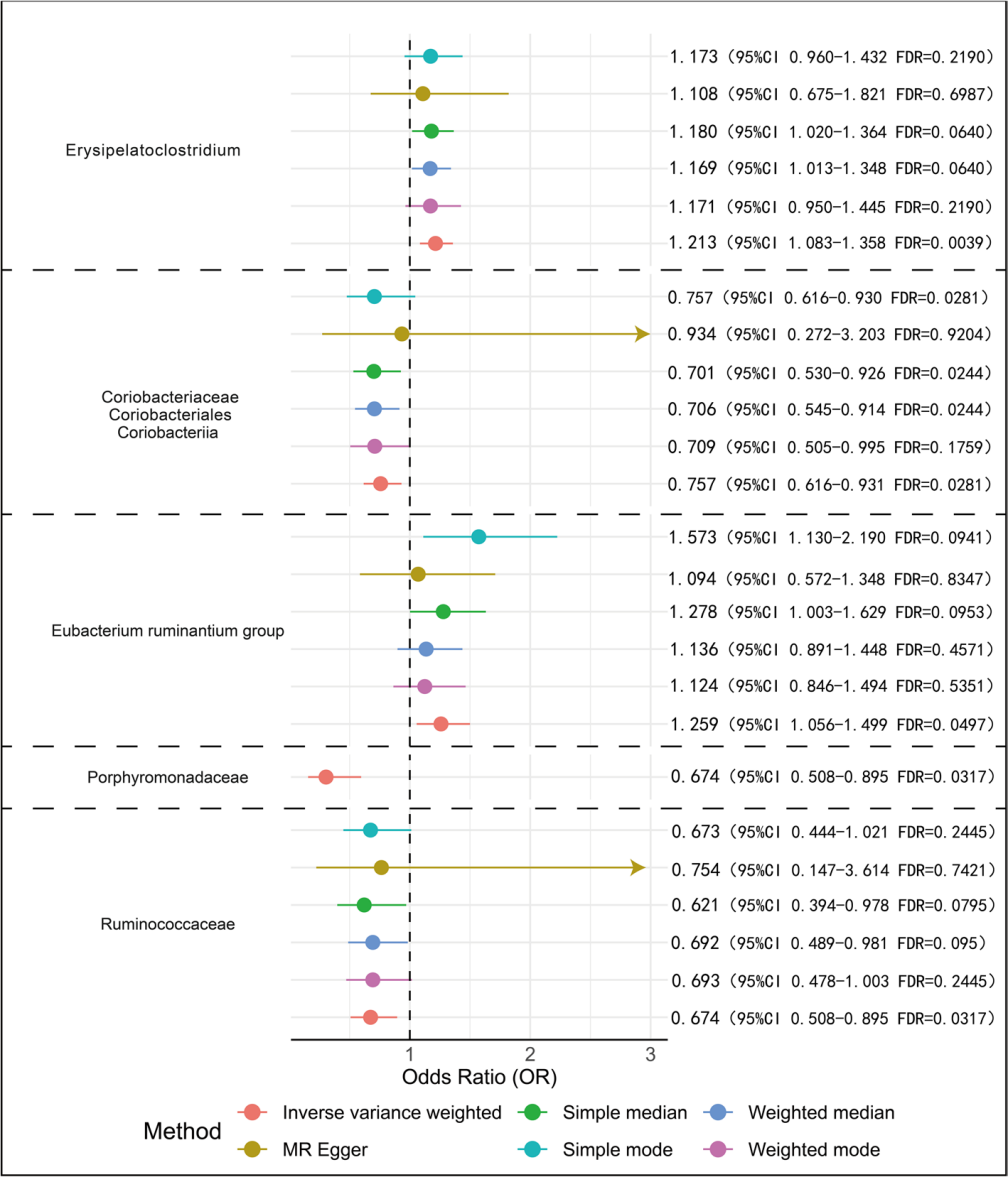
The Link Between Gut Microbiota and Breast Cancer in Europeans. The lines represent the 95% confidence interval (CI) for the odds ratio, with arrows indicating when the CI bounds exceed the x-axis range. “FDR” stands for “False Discovery Rate”. Only the IVW method was used for Porphyromonadaceae because the selection process yielded only two instrumental variables, which were suitable exclusively for IVW and not applicable for other methods

Based on the IVW method, in the European population, Erysipelatoclostridium was observed to have a positive association with breast cancer risk, with an OR of 1.21 (95% CI 1.083–1.358, FDR = 0.0039) (Fig. 3). Coriobacteriales, Coriobacteriaceae, and Coriobacteriia were inversely associated with breast cancer risk, with an OR of 0.757 (95% CI 0.616–0.930, FDR = 0.0281) (Fig. 3). All findings met the statistical significance threshold of FDR < 0.05 (Fig. 3).

Based on the IVW method, in the East Asian population, Porphyromonadaceae had an inversely association with breast cancer risk, with an OR of 0.304 (95% CI 0.155–0.596, FDR = 0.0005), and Ruminococcaceae also showed an inversely association with breast cancer risk, with an OR of 0.674 (95% CI 0.508–0.895, FDR = 0.03173). The *Eubacterium ruminantium* group showed a positive association with breast cancer risk, with an OR of 1.259 (95% CI 1.056–1.499, FDR = 0.0497).

**Fig. 4:**
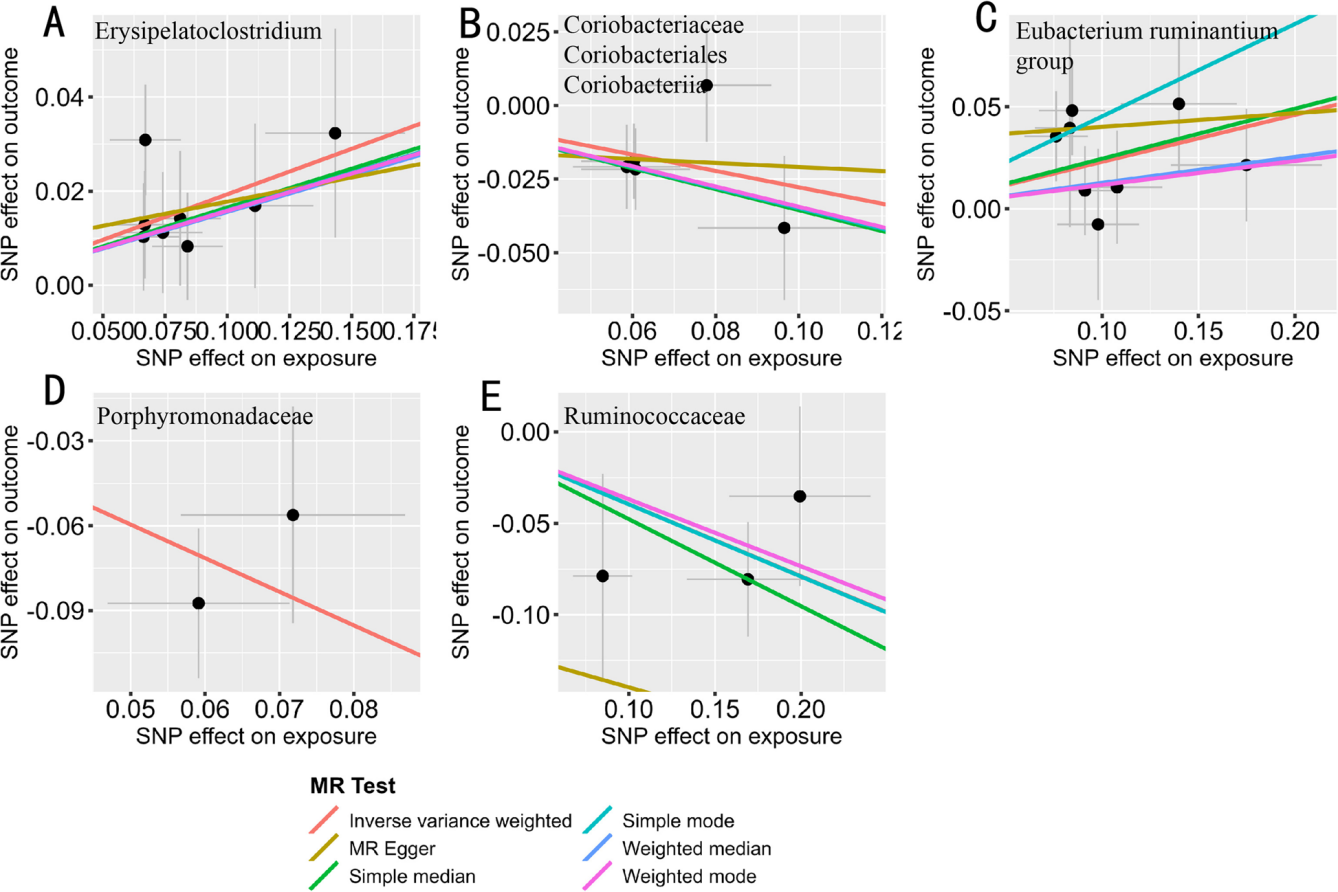
The Scatter Plot shows the effect of SNPs on the gut microbiome and breast cancer, with gray error bars representing the 95% confidence interval of the effect

Regardless of being in European or East Asian populations and despite the differences in OR values and corresponding FDR values, the MR methods used consistently demonstrated consistent causal estimates between gut microbiota and breast cancer risk (Fig. 4). However, due to the limited number of IVs for the gut microbiota families Porphyromonadaceae and Ruminococcaceae, not all MR methods could be applied, and their results may be unstable.

## Discussion

In this study, We found association in the European population between the class Coriobacteriia, the order Coriobacteriales, the family Coriobacteriaceae, and the genus *Erysipelatoclostridium* and breast cancer. IVs for Coriobacteriia, Coriobacteriales, and Coriobacteriaceae, selected based on a statistical threshold of *p*-value less than 5 × 10–6, were completely consistent. These taxa may be associated with breast cancer risk through common biological pathways. It is important to note that due to the hierarchical nature of taxonomic classification, the observed associations at different taxonomic levels may reflect a common underlying biological mechanism. Therefore, the associations observed for Coriobacteriia, Coriobacteriales, and Coriobacteriaceae may not represent independent effects, but rather indicate a single type of microbiota influencing breast cancer risk.

*Erysipelatoclostridium* is an anaerobic bacterium associated with intestinal health and disease. Iadsee et al. showed that levels of *Erysipelatoclostridium ramosum* were significantly higher in colorectal cancer patients compared to the healthy population [24]. Cai et al. found that its metabolic product, Ptilosteroid A, is associated with radiation-induced intestinal injury, demonstrating significant value in predicting radiation-induced intestinal damage [25]. Our study suggests an association between *Erysipelatoclostridium* and breast cancer, though the specific mechanisms remain unclear.

Some bacteria within the Coriobacteriia family can participate in estrogen metabolism, primarily by metabolizing soy isoflavones such as daidzein and genistein, which are converted in the body into compounds with estrogenic activity [26]. Therefore, they may play an essential role in the development of hormone-dependent cancers, such as breast and prostate cancer, by regulating estrogen levels and affecting the risk and progression of cancer.

In East Asian populations, we discovered that Porphyromonadaceae and Ruminococcaceae are negatively correlated with the risk of breast cancer, while the *Eubacterium ruminantium* group is positively correlated. This finding significantly differs from the findings in European populations. This difference reflects the significant disparities in genetic backgrounds, dietary habits, lifestyles, and environmental factors among various populations. These factors can significantly impact the composition of the gut microbiota, influencing the risk of developing breast cancer. These results underscore the importance of considering population-specific factors when studying the relationship between the gut microbiome and breast cancer. Porphyromonadaceae and Ruminococcaceae, known for their relation to gut health and inflammation regulation, are particularly interesting. Some members of the Porphyromonadaceae family have been associated with intestinal inflammation and the maintenance of gut barrier function [27], although research in this area remains relatively sparse. The Ruminococcaceae family is noted for producing short-chain fatty acids (SCFAs) [28], including butyrate, propionate, and acetate. SCFAs are crucial for promoting the expression of tight junction proteins, reducing intestinal permeability, preventing the transmembrane transport of harmful substances and pathogens, and regulating the intestinal immune response [29]. This phenomenon leads to anti-inflammatory and immune tolerance effects and activates G-protein-coupled receptors (such as GPR41 and GPR43) that regulate the host’s energy balance and metabolism [30]. Thus, the Ruminococcaceae family and its metabolic products, short-chain fatty acids (SCFAs), have shown great potential in the prevention and treatment of various diseases. This includes improving gut health, reducing the risk of inflammatory bowel disease (IBD) and colorectal cancer [31, 32], as well as combating obesity and type 2 diabetes [29]. Consequently, they have become a significant focus in current microbiome research.

Notably, the instrumental variables for the gut microbiota Porphyromonadaceae and Ruminococcaceae are too few, limiting the statistical power of our analysis. Although these results are statistically significant, their interpretation should be approached with caution. Conversely, the Eubacterium ruminantium group has a sufficient number of instrumental variables, making the statistical results related to its association with breast cancer risk more reliable.

This study aimed to use MR to reveal the relationship between gut microbiota and breast cancer in European and East Asian populations. We used GWAS summary data from the MiBioGen consortium, the largest GWAS dataset on microbiomes to date, with breast cancer data from FinnGen and Biobank Japan, providing a solid foundation for our analysis. Despite differences between the two populations, our findings suggest potential associations between specific microbiota and breast cancer.

This study has some limitations. Despite setting a significance threshold that is not very stringent (*P* < 5 × 10^–6^), including a relatively small number of instrumental variables limited our statistical analysis power and could potentially lead to false positive results. To address this issue, we implemented multiple testing corrections by calculating the FDR to enhance the reliability of our findings. Furthermore, although MR analysis inherently addresses confounding issues, it remains susceptible to pleiotropic effects. Our study applied sensitivity analysis methods such as MR-Egger and MR-PRESSO to mitigate genetic pleiotropy. The consistent results across various MR analysis methods indicate the robustness of our findings.

Nevertheless, a significant limitation is the absence of stratification based on specific breast cancer subtypes, such as HER-2, ER, and PR expression statuses. Additionally, our study did not adjust for patient demographics, dietary factors, or other environmental factors that are closely associated with both exposure and outcome. Considering the different interactions between gut microbiota and various breast cancer subtypes, as well as the potential confounding effects of these unadjusted factors, the generality of our findings may be limited. This aspect should be considered when interpreting our results, and future genetic studies should delve deeper into these specific subtypes as well as the potential confounding effects of these unadjusted factors to provide a more comprehensive understanding. Other limitations include the cross-sectional nature of GWAS data, complicating the determination of a temporal causal relationship between gut microbiota changes and breast cancer onset. Extra caution is required when interpreting the summary data provided by GWAS, as it does not account for the complexity and nuances arising from individual differences. Although MR is a powerful tool for exploring causal relationships between genetic variations and health outcomes, it must be acknowledged that it has its own limitations. Pleiotropy and sample size constraints may lead to biased results, and genetic heterogeneity may affect the general applicability of the study findings.

Despite these limitations, an increasing body of evidence suggests that exploring the gut microbiome offers new perspectives in unveiling the pathogenesis of breast cancer and enhancing the predictive accuracy of existing risk assessment methodologies [14, 33, 34]. The findings of this study have significant potential clinical implications. First, the association between specific gut microbiota and breast cancer risk could serve as new biomarkers for early detection and prevention of breast cancer. By monitoring changes in the composition of gut microbiota, high-risk individuals may be identified, enabling early intervention. Second, understanding the relationship between these microbiota metabolites and breast cancer can aid in developing new therapeutic strategies. For example, the role of Erysipelatoclostridium and its metabolite Ptilosteroid A in predicting radiation-induced intestinal injury suggests its potential in breast cancer treatment. Research has reported that identifying gut microbiome characteristics can provide critical information for predicting the efficacy and safety of chemotherapy in breast cancer patients, playing a role in developing personalized treatment strategies [35]. Additionally, our results indicate that the relationship between gut microbiota and breast cancer varies among different populations, highlighting the importance of personalized treatment.

## Conclusions

In summary, our study provides new insights into the relationship between the gut microbiota of European and East Asian populations and breast cancer. The identified microbiota may represent potential biomarkers or therapeutic targets for breast cancer. Future research is needed to validate these findings and reveal their specific biological mechanisms.

## Data Availability

Summary data on gut bacteria were obtained from the MiBioGen consortium’s genome-wide association meta-analysis, accessible at https://mibiogen.gcc.rug.nl/. The breast cancer GWAS data for the European population were sourced from the FinnGen consortium’s R10 release, available for download from https://storage.googleapis.com/finngen-public-data-r10/summary_stats/R10_manifest.tsv/. The breast cancer GWAS data for the East Asian population were sourced from the Biobank Japan project, available for download from the IEU database (GWAS ID: bbj-a-160; https://gwas.mrcieu.ac.uk/).
